# RNA-Seq Uncovers SNPs and Alternative Splicing Events in Asian Lotus (*Nelumbo nucifera*)

**DOI:** 10.1371/journal.pone.0125702

**Published:** 2015-04-30

**Authors:** Mei Yang, Liming Xu, Yanling Liu, Pingfang Yang

**Affiliations:** 1 Key Laboratory of Plant Germplasm Enhancement and Speciality Agriculture, Wuhan Botanical Garden, Chinese Academy of Sciences, Wuhan, Hubei, China; 2 Key Laboratory of Aquatic Plant and Watershed Ecology, Wuhan Botanical Garden, Chinese Academy of Sciences, Wuhan, Hubei, China; Chinese Academy of Fishery Sciences, CHINA

## Abstract

RNA-Seq is an efficient way to comprehensively identify single nucleotide polymorphisms (SNPs) and alternative splicing (AS) events from the expressed genes. In this study, we conducted transcriptome sequencing of four Asian lotus (*Nelumbo nucifera*) cultivars using Illumina HiSeq2000 platform to identify SNPs and AS events in lotus. A total of 505 million pair-end RNA-Seq reads were generated from four cultivars, of which 86% were mapped to the lotus reference genome. Using the four sets of data together, a total of 357,689 putative SNPs were identified with an average density of one SNP per 2.2 kb. These SNPs were located in 1,253 scaffolds and 15,016 expressed genes. A/G and C/T were the two major types of SNPs in the Asian lotus transcriptome. In parallel, a total of 177,540 AS events were detected in the four cultivars and were distributed in 64% of the expressed genes of lotus. The predominant type of AS events was alternative 5’ first exon, which accounted for 41.2% of all the observed AS events, and exon skipping only accounted for 4.3% of all AS. Gene Ontology analysis was conducted to analyze the function of the genes containing SNPs and AS events. Validation of selected SNPs and AS events revealed that 74% of SNPs and 80% of AS events were reliable, which indicates that RNA-Seq is an efficient approach to uncover gene-associated SNPs and AS events. A large number of SNPs and AS events identified in our study will facilitate further genetic and functional genomics research in lotus.

## Introduction

Lotus belongs to Nelumbonaceae, a small plant family with only one genus, *Nelumbo*, and two species: *N*. *nucifera* (distributed in Asia, Australia, Russia) and *N*. *lutea* (distributed in eastern and southern North America) [1]. It is an aquatic perennial, and has ornamental, edible and medicinal value, as well as religious significance. Because of its beautiful flowers and edible rhizomes and seeds, lotus has been cultivated as ornamental or vegetable plant for more than 7,000 years throughout Asia. Nearly every part of the lotus plant including buds, flowers, anthers, stamens, fruits, leaves, stalks, rhizomes and roots have been used as herbal medicines for treatment of cancer, heart problems, hypertension and insomnia [2].

In the recent past, the genome of lotus had been sequenced and assembled with Illumina and 454 technologies [3], which marked the beginning of a new era on genetic and genomic studies of lotus. Although a large set of microsatellite markers have been developed [4] and could be used for linkage mapping and association study, there are still no sufficient markers for genome wide association studies (GWAS). Single nucleotide polymorphism (SNP) markers could meet the needs on both marker density and genome coverage, and have been applied in linkage mapping and GWAS in many species, for instance, *Arabidopsis* [5, 6], rice [7, 8], maize [9–11], soybean [12], sunflower [13, 14] and *Cucurbita* [15]. In lotus, using the restriction-site associated DNA sequencing (RAD-Seq) technologies, 4,098 SNPs have been developed for the F_1_ population derived from a cross between *N*. *nucifera* ‘China Antique’ and *N*. *lutea* ‘AL1’ [16]. However, the number of SNPs is yet limited for QTL analysis, fine mapping and GWAS in lotus. RNA-Seq on Illumina platform could generate redundant transcriptome sequences with high read depth and is a powerful way of identifying large scale SNPs from transcribed regions in the genomes [17–20]. Large number of SNPs have been developed by transcriptome analysis in several species, including sunflower [21], sabaigrass [22], melon [23], pepper [24], onion [20] and peach [25]. However, SNPs discovery in transcriptome data by RNA-Seq has not been reported in *Nelumbo* till present.

Additionally, RNA-Seq provides huge data sets for deep exploration of alternative splicing (AS) events [26]. AS is considered to be an important posttranscriptional regulatory mechanism for modulating gene expression and functional complexity in higher eukaryotes. It was estimated that AS events could produce premature termination codons, and alter the coding sequence [27, 28]. AS is commonly found in plant species. RNA-Seq suggested that 61% of *Arabidopsis* genes [29], 21.2–33% of rice genes [30] and 63% of soybean genes [31] are subjected to AS. There are four major types of AS: intron retention (IR), exon skipping (ES), alternative 5′ splice sites, and alternative 3′ splice sites [32–34]. IR is more frequent in plants such as *Arabidopsis* and rice, and ES only accounts for a small portion of AS [29, 33, 35]. The mechanisms regulating AS are still poorly understood, and their complexity is attributed to the combination of numerous regulation factors: including splicing factors, cis-regulatory elements, and RNA secondary structures [36]. Although the AS events have been identified from expressed sequence tags (ESTs) in lotus [37], the landscape of AS has not been explored from lotus RNA-Seq transcriptome data.

In this study, RNA-Seq were conducted on leaves and rhizomes of four Asian lotus cultivars. Based on extensive data analyses, we have identified SNPs and AS events from transcribed regions. These SNPs will provide useful resources for population genetic study, genetic linkage analysis and genome-wide association studies. Identified AS events could reveal the changes in gene structure and genomic features of lotus. Our results will facilitate an in-depth understanding of genetic and genomic research in lotus species.

## Materials and Methods

### Plant materials

Four cultivars, ‘Bai Ge’ (‘BG’), ‘Winter Red 1’ (‘WR1’), ‘Zhou Ou’ (‘ZO’) and ‘Red Lingxiao’ (‘RL’), of *N*. *nucifera*, were used in this study. Both ‘BG’ and ‘ZO’ are temperate cultivars with enlarged rhizomes. To the contrast, ‘WR1’ and ‘RL’ are tropical cultivars with thin rhizomes [38]. These four cultivars have been conserved as rhizome at Wuhan Botanical Garden of the Chinese Academy of Sciences (N30°32′44.02″, E114°24′52.18″), Hubei Province, China for many years. Leaves were collected from ‘BG’ and ‘WR1’ at the initial developmental stage and the fast developing stage of flower buds, respectively. Rhizomes were collected from ‘ZO’ and ‘RL’ at stolon, middle swelling, and later swelling stage of rhizome development, respectively. All samples were immediately transferred to liquid nitrogen, and stored subsequently at -80°C until RNA extraction. The samples of each stage were collected from three comparable plants for replications.

### RNA isolation, library construction, and sequencing

Total RNA was extracted using the GREENspin RNA reagent (ZP411, Zoman Biotechnology, Beijing, China) according to the manufacturer’s protocol, and treated with RNase-free DNase I (Takara, Dalian, China) to remove genomic DNA contamination. RNA integrity was evaluated with a 1.0% agarose gel stained with ethidium bromide (EB). Thereafter, the quality and quantity of RNA were assessed using a NanoPhotometer spectrophotometer (IMPLEN, CA, USA) and an Agilent 2100 Bioanalyzer (Agilent Technologies, CA, USA). The RNA integrity number (RIN) was greater than 8.0 for all samples. For each developmental stage of the four cultivars, the RNA samples from the three individual repeats were pooled together in equal amounts to generate one mixed sample. These mixed RNA samples were subsequently used in cDNA library construction and Illumina sequencing.

A total amount of 3 μg RNA per sample was used to construct cDNA library. The library was generated using NEBNext Ultra RNA Library Prep Kit for Illumina (NEB, USA) following manufacturer’s recommendations. Briefly, poly(A) mRNA was purified from total RNA using oligo(dT)-attached magnetic beads. The mRNA was then cleaved into small fragments by exposure to divalent cations under an elevated temperature in NEBNext first strand synthesis reaction buffer (5X). These fragments were used to synthesize first-strand cDNA using random hexamer primer and M-MuLV reverse transcriptase (RNase H). Second-strand cDNA synthesis was subsequently performed using DNA polymerase I and RNase H. Remaining overhangs were converted into blunt ends via exonuclease/polymerase activities. After adenylation of 3’ ends of DNA fragments, NEBNext adaptor with hairpin loop structure was ligated to prepare for hybridization. In order to select cDNA fragments of preferentially 150–200 bp in length, the library fragments were purified with AMPure XP system (Beckman Coulter, Beverly, USA). Then 3 μL USER Enzyme (NEB, USA) was used with size-selected, adaptor-ligated cDNA at 37°C for 15 min followed by 5 min at 95°C before PCR. Then PCR was performed with Phusion high-fidelity DNA polymerase, Universal PCR primers and index (X) primer. At last, PCR products were purified (AMPure XP system) and library quality was assessed on the Agilent Bioanalyzer 2100 system.

The clustering of the index-coded samples was performed on a cBot Cluster Generation System using TruSeq PE Cluster Kit v3-cBot-HS (Illumina) according to the manufacturer’s instructions. After cluster generation, the library preparations were sequenced on an Illumina Hiseq 2000 platform and 100 bp paired-end reads were generated. All clean sequence reads data were deposited in Sacred Lotus Genome Annotation Project (SGAP, http://202.127.146.141/genome_download/rna_seq/).

### Reads mapping to the reference genome and transcript annotation

The raw reads were cleaned by removing adapter sequences, reads containing ploy-N, and low-quality sequences (Q < 20). Clean reads were aligned to the reference genome sequence using the program Tophat v2.0.9 [39]. The tolerance parameters were the default settings, allowing mismatches of no more than two bases. For annotations, all novel genes were searched against the Nr database using BLASTx with 10^–5^ as E-value cut-off point and sequences with the highest similarities were retrieved.

### Identification of SNP and AS events

Picard-tools v1.96 (http://sourceforge.net/projects/picard/files/picard-tools/1.96/) and samtools v0.1.18 [40] were used to sort, mark duplicated reads and reorder the bam alignment results of each sample. GATK2 software [41] was used to perform SNP calling. Filtering thresholds were set as: consensus quality is no less than 30 and coverage is no less than 10. In order to get more reliable SNPs, those that were identified in at least two stages for each cultivar were regarded as the SNPs for this cultivar.

The AS events were classified into seven basic types, including alternative 5’ first exon, alternative 3’ last exon, exon skipping (ES), multi-exon skipping (MES), intron retention (IR), multi-intron retention (MIR), and alternative exon ends (AE), by the software Asprofile v1.0 [42]. The AS events that were identified in at least two stages for each cultivar were regarded as the stable ASs for this cultivar.

### Gene Ontology enrichment analysis of expressed genes

Gene Ontology (GO) analysis was performed to annotate genes which contained SNPs and AS events. GO enrichment analysis of genes was implemented by the GOseq R package, in which gene length bias was corrected. GO terms with corrected *P* value less than 0.05 were considered significantly enriched by differential expressed genes. The GO annotations were functionally classified by WEGO software [43] for gene function distributions.

### Validation of SNPs and AS events

In order to validate the accuracy of SNPs prediction, 53 SNPs were randomly selected for SNP validation using DNA as templates. Except for the four cultivars sampled for RNA-Seq, other five lotus cultivars, ‘Yehong Lian’, ‘Jianxian 17’, ‘Luming Lian’, ‘AL1’, and ‘Golden Bird’ were also used to validate SNPs. Primers were designed to amplify the flanking sequence of selected SNPs using Primer 3 (http://bioinfo.ut.ee/primer3-0.4.0/). The amplified PCR products were sequenced by Sanger method and analyzed by BioEdit v 7.0.5.3 (http://www.mbio.ncsu.edu/BioEdit/bioedit.html).

Seven AS genes were chosen to validate the accuracy of the AS prediction in this study. Fragments containing the AS events were amplified using RT-PCR, and were then submitted for sequencing. The sequencing results were analyzed by BioEdit v7.0.5.3. The sequences of primers for SNP and AS validation were listed in S1 Table.

## Results and Discussion

### Overview of the RNA-Seq data

Sequencing on Illumina HiSeq2000 platform was conducted to generate short sequence reads of expressed sequences. After filtering with NGS QC Toolkit [44], a total of 72.68, 74.85, 184.93, and 172.57 million high-quality reads in length of 100 bp were generated from ‘BG’, ‘WR1’, ‘ZO’, and ‘RL’, respectively. The short reads of RNA-Seq data from four cultivars had aligned onto reference genome of *N*. *nucifera* ‘China Antique’ [3]. There are 85.4%, 84.4%, 90.7%, and 86.0% of the short reads from ‘BG’, ‘WR1’, ‘ZO’, and ‘RL’, mapped on reference genome (Table 1). The mapping ratio of ‘ZO’ is slightly higher than that of the other three cultivars, which may be due to the close genetic relationship between ‘ZO’ and ‘China Antique’. This observation is supported by the result that ‘ZO’ is phylogenetically closer to ‘China Antique’ [45].

**Table 1:** Statistics of RNA-Seq clean reads and mapped reads ratio against in the lotus reference genome.

	No. of clean reads (million)	No. of mapped reads (million)	Mapped reads ratio (%)
‘BG’	72.68	61.68	85.42
‘WR1’	74.85	63.21	84.44
‘ZO’	184.93	167.75	90.71
‘RL’	172.57	148.48	86.04

**Fig 1:**
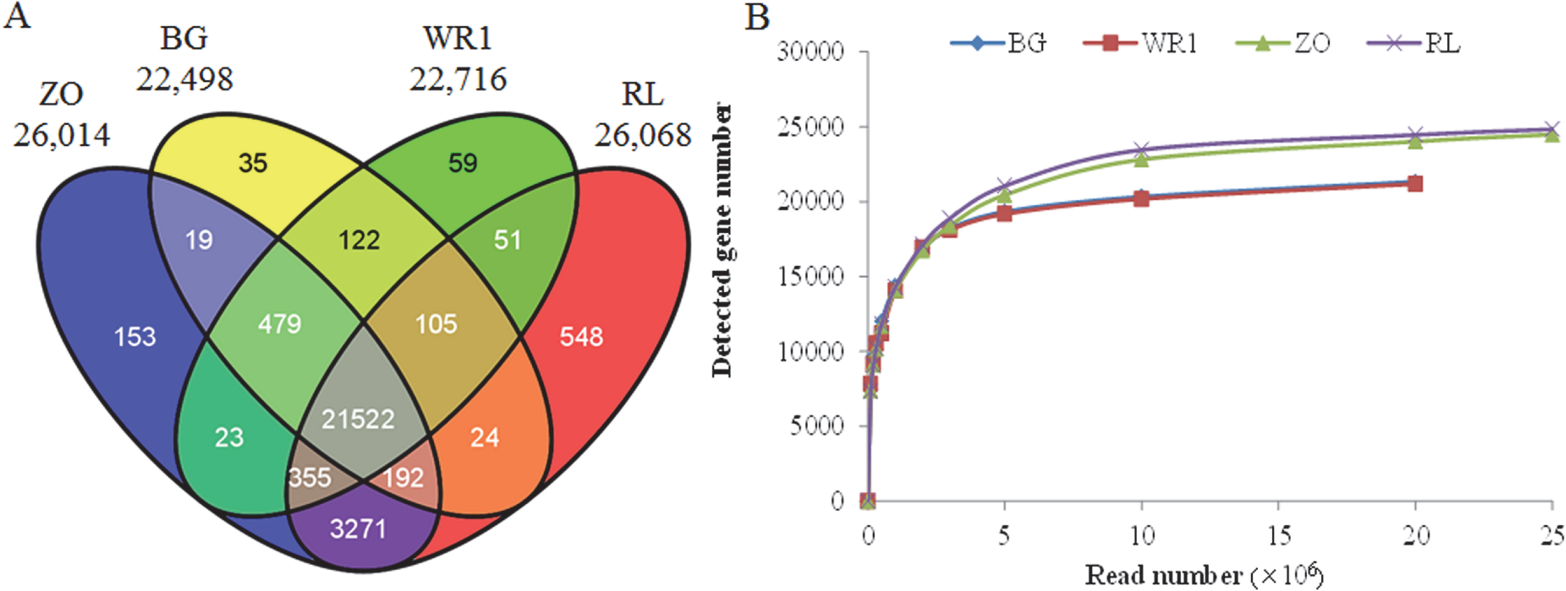
Summary of gene number identified from the Asian lotus transcriptome. (A) Venn diagram of genes expressed in the four cultivars. (B) Correlations between detected genes number and reads number in the four cultivars.

A total of 29,400 genes were assembled by mapping the reads from the four samples to the reference genome. While 26,685 genes were previously predicted in the lotus reference [3], the rest genes were novel transcripts. Of these, 22,498, 22,716, 26,014 and 26,068 genes were identified from ‘BG’, ‘WR1’, ‘ZO’, and ‘RL’, respectively. The majority of these genes were commonly detected in the four cultivars, and more than three thousand genes were detected in ‘ZO’ and ‘RL’ than in ‘BG’ and ‘WR1’ (Fig 1A). Such differences on gene number among them were due to the different expressed genes between leaf and rhizome but not caused by the sequencing reads of the four cultivars, which was proved by the correlations between detected gene number and read number (Fig 1B). Ming et al. [3] also identified more expressed genes in rhizome than in leaf.

### Identification, distribution and functional analysis of SNP

**Fig 2:**
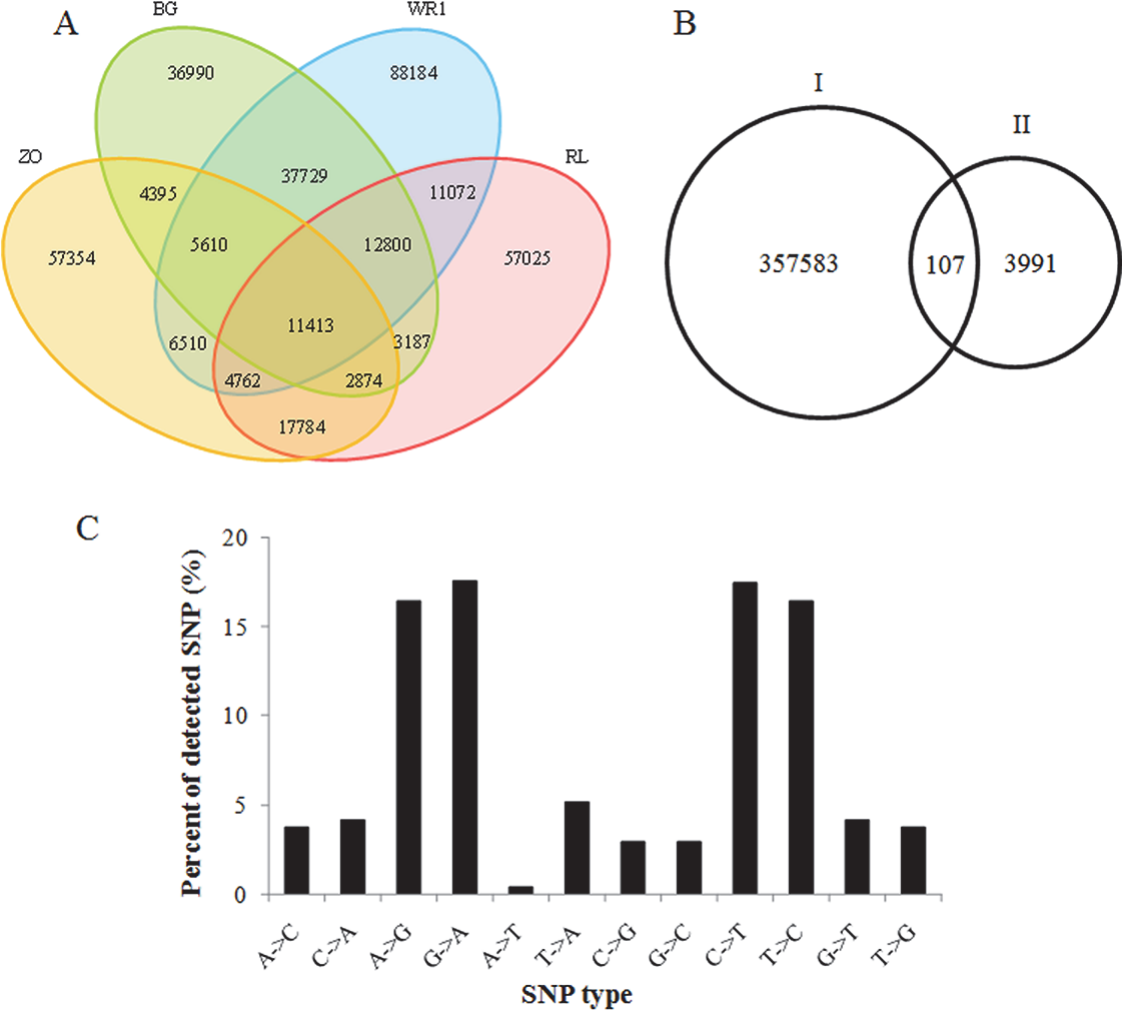
Number and type of SNPs identified from the Asian lotus transcriptome. (A) Venn diagram of SNPs discovered from the four cultivars. (B) Comparison of SNP number between our study (I) and the previous study of Zhang et al. [16] who discovered SNP by RAD-Seq technology (II). (C) Summary of SNP types identified from the Asian lotus transcriptome.

Putative SNPs were predicted from four Asian lotus cultivars based on read depth and quality score of alignment results. A total of 114,998, 178,080, 110,702, and 120,917 putative SNPs with high quality were predicted in the ‘BG’, ‘WR1’, ‘ZO’, and ‘RL’, respectively. Among them, 36,990, 88,184, 57,354, and 57,025 SNPs were specific for ‘BG’, ‘WR1’, ‘ZO’, and ‘RL’, respectively. And 11,413 SNPs were commonly detected in all four cultivars. The number of commonly SNPs detected between ‘BG’ and ‘WR1’ was the largest, but that between ‘BG’ and ‘ZO’ was the smallest (Fig 2A). This might be caused by the closer relationship of ‘BG’ with ‘ZO’ than with ‘WR1’. Using the four sets of data together, a total of 357,689 putative SNPs were identified from all four libraries, and only 107 SNPs had been detected in Zhang et al. [16] who discovered SNP by RAD-Seq technology (Fig 2B). The lower similarity between the two studies was possibly due to the fact that the SNPs in our study were identified among the cultivars of *N*. *nucifera*, while the ones in Zhang et al. [16] were detected between the cultivars of *N*. *nucifera* and *N*. *lutea*. An average density of SNP is one SNP per 2.2 kb, which is higher than those reported in pepper with one SNP per 2.5 kb [24] and in peach with one SNP per 5.7 kb [25], respectively. However, the observed SNP density in lotus is lower than that in onion with an average of one SNP per 1.7 kb [20], sunflower with one SNP per 0.16 kb [21], and sabaigrass with one SNP per 0.34 kb [22].

Among the SNPs, A/G and C/T were the two most abundant and evenly present, with each accounting for 34% of all SNPs, respectively (Fig 2C). The frequency of SNP types in the lotus genome is in close agreement with the patterns observed in red pepper [18], peach [25], and sabaigrass [22]. The SNPs were classified into several categories based on their locations in the genome, including intergenic, downstream, exon, intron, and upstream. As shown in Table 2, of the all putative SNPs, 116,653 SNPs (32.6%) were identified in intergenics, and 88,748 (24.8%) were detected in introns, while only 32,013 SNPs (8.9%) in the exons, which were lowly represented.

**Table 2:** Classification of identified SNPs.

SNP classification	SNP number	Percent (%)
Intergenic	116653	32.6
Downstream	54391	15.2
Exon	32013	8.9
Intron	88748	24.8
Upstream	35628	10.0

Intergenic SNPs were identified from regions between genes, while Downstream and Upstream represents SNPs identified from regions of downstream and upstream of the genes.

**Fig 3:**
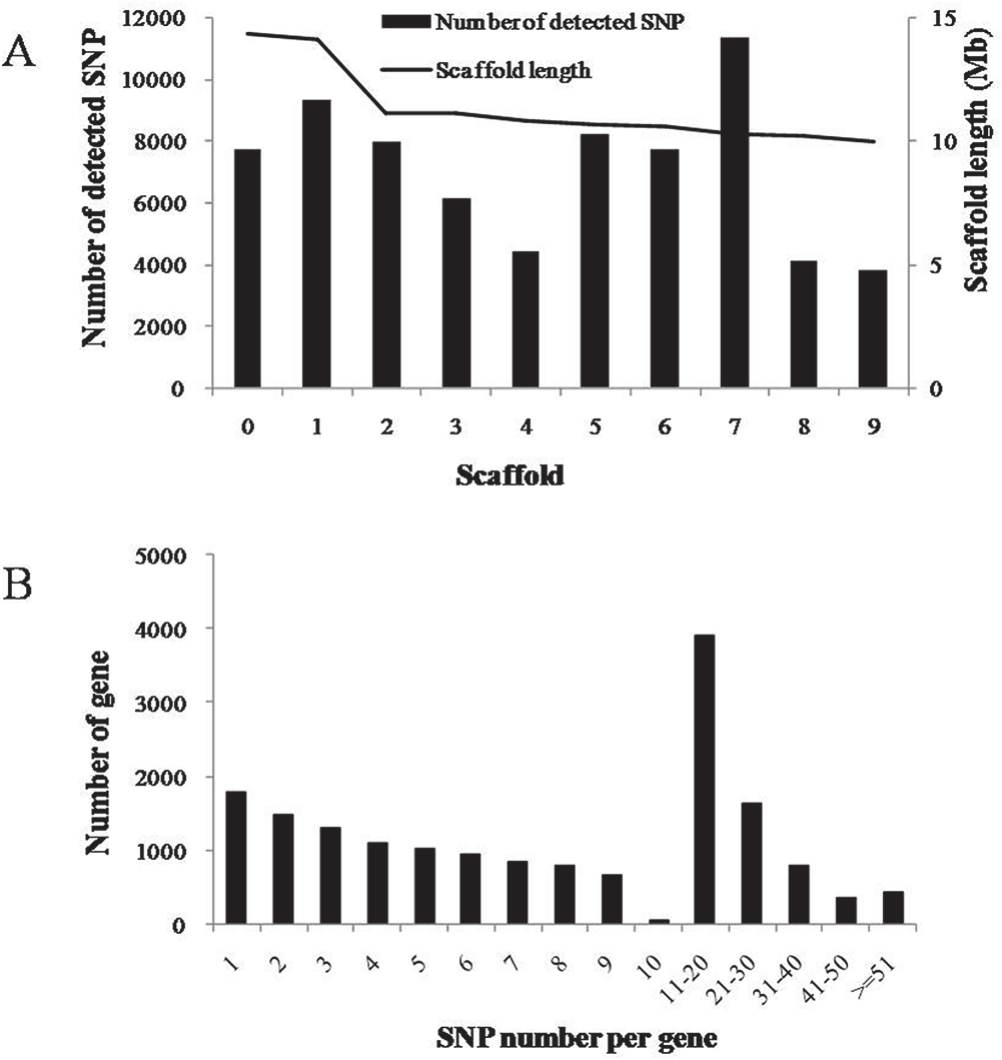
SNP distribution among scaffolds and genes. (A) Distribution of expressed SNPs on the top ten scaffold. (B) Number of SNP per gene.

SNP distribution among genes is important when considering the marker density and genome coverage using SNP marker [22–24], especially when these SNPs were used for linkage map construction. Here we analyzed SNPs distribution among all genome scaffolds and expressed genes (Fig 3). The SNPs identified in the present study were found on the 1,253 scaffolds (data not shown), about 285.5 SNPs per scaffold on average. Less than 20% of SNP and SNP genes were located on the top ten scaffolds. The scaffold_7 and scaffold_1 had the largest number of SNPs, 11,327 and 9,325 SNPs, respectively. The distribution of these SNPs on the scaffolds was regardless of the length of scaffold (Fig 3A). All the SNPs were distributed in 15,016 genes. On average, 23.8 SNPs per gene were identified. Among these genes, those with no more than 10 SNPs occupied 56.46% of total genes (Fig 3B).

**Fig 4:**
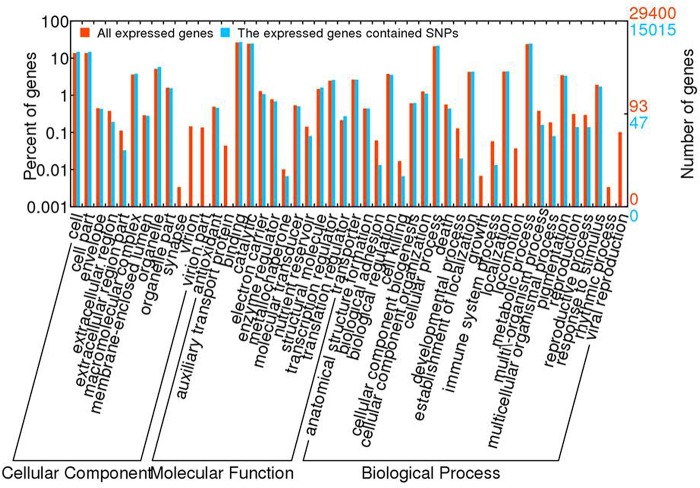
GO analysis of all the expressed genes in lotus and the genes contained SNP.

GO annotation was performed for the genes containing SNPs using all lotus genes as the background (Fig 4). In total 7,563 expressed genes containing SNPs were assigned with one or more GO terms. For biological process, genes involved in the metabolic process and cellular process were overrepresented. For molecular function, binding and catalytic activity were the most represented GO terms. Regarding to the cellular component, the major categories were cell and cell part. SNP was not detected in the genes in growth, locomotion, rhythmic process and viral reproduction process in biological process category. No SNP was contained in the genes in auxiliary transport protein in molecular function category, and no SNP was detected in the genes in synapse, virion and virion part in cellular component category, either.

### Identification and annotation of AS events

**Fig 5:**
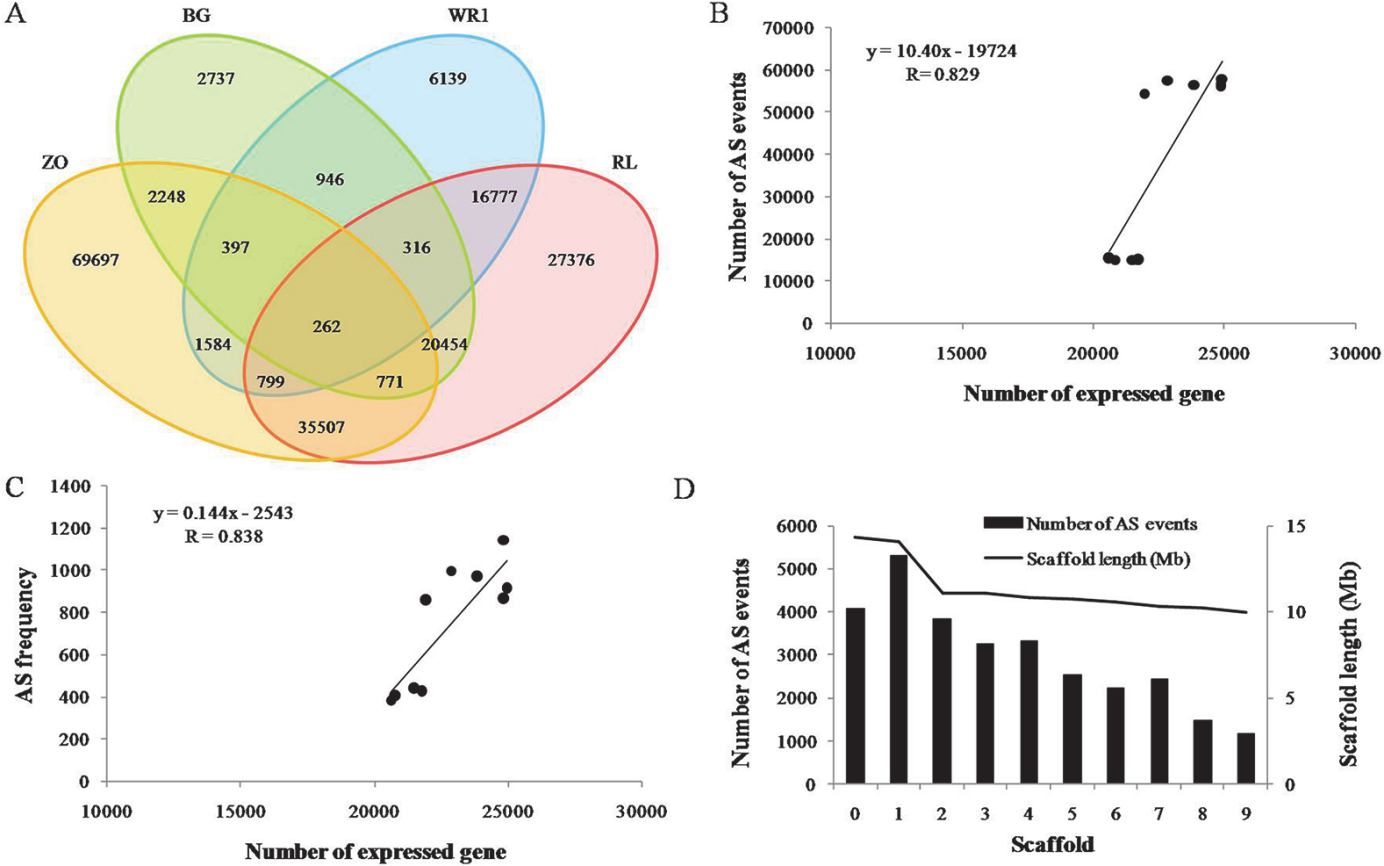
Numbers of AS events and distribution in the lotus Genome. (A) Venn diagram of AS events discovered from the four datasets. (B) Correlations of AS event number with expressed gene number. (C) Correlations of AS frequency with expressed gene number. (D) Distribution of AS events in the lotus genome.

A total of 177,540 AS events were identified in the four data sets, and were distributed in 18,842 genes, which accounted for 64% of the total expressed genes [3, 38]. The ratio is similar with those reported in *Arabidopsis* [29] and soybean [31], but much higher than that reported in rice [30]. The ratios for these three species are 61%, 63%, and 21.2–33% respectively. There were 28,131, 27,994, 111,262, and 109,462 AS events from ‘BG’, ‘WR1’, ‘ZO’, and ‘RL’, respectively, which were identified in 5,632, 5,353, 17,137, and 17,955 genes (Table 3). There were 2,737, 6,139, 69,697, and 27,376 AS events specific for ‘BG’, ‘WR1’, ‘ZO’, and ‘RL’, respectively. Only 262 AS events were commonly detected in all four cultivars (Fig 5A). Such a great difference on AS events detected in the four libraries might be due to the different sequencing depth of the four cultivars (Table 1). To eliminate the bias effect of the sequencing depth, we calculated the AS frequency (number of AS events / total read number) for each cultivar. We found that the AS frequency was also higher in ‘ZO’ and ‘RL’ than that in ‘BG’ and ‘WR1’ (Table 3). This suggested that the AS events detected in rhizome were higher than those detected in leaves. In general, more AS was found in the rapidly developing tissues and functionally complex tissues [31]. Therefore rhizomes might be undergoing more vigorous metabolism than leaves at the given sampling stages, consequently more genes expressed than leaves (Fig 1A). A correlation analysis using data from all samples also showed that the AS events and their frequency were highly correlated with the expressed gene number (Fig 5B and 5C). It is proposed that AS is an important factor in regulating gene expression [35]. The distribution of the transcripts containing AS events across the top ten scaffold of the lotus genome are shown in Fig 5D. Less than 20% of the AS events and AS genes are located on scaffolds 0–9, regardless of the length of scaffold.

**Table 3:** Statistics of AS discovered from RNA-Seq data.

	Number of AS events	Number of AS genes	AS frequency
‘BG’	28131	5632	387.1
‘WR1’	27994	5353	374.0
‘ZO’	111262	17137	601.6
‘RL’	109462	17955	634.3
Total	177540	18842	

**Fig 6:**
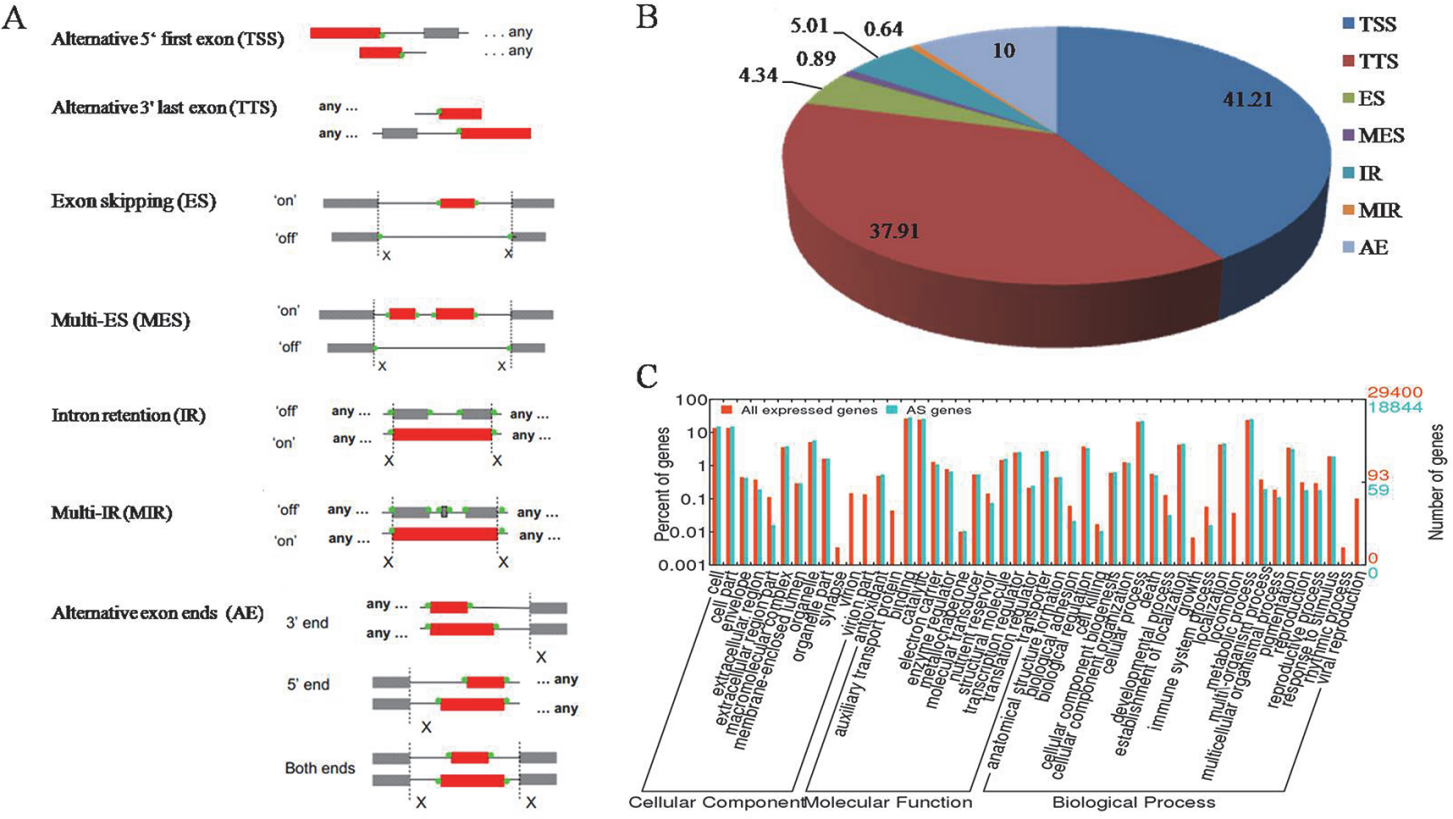
Statistics of the different AS events and GO analysis of AS genes. (A) Diagram of different types of AS events. (B) Proportion of different types of AS events. (C) GO analysis of AS genes using all expressed genes for lotus as the background.

Seven types of AS events were detected in the Asian lotus transcriptome, including alternative 5’ first exon, alternative 3’ last exon, ES, MES, IR, MIR, and AE (Fig 6A). Out of the 177,540 AS events identified, alternative 5’ first exon represented 41.2% and was the most abundant type, followed by alternative 3’ last exon (37.9%), AE (10.0%), IR (5.0%), and ES (4.3%). MES and MIR were rare and accounted only for 0.9% and 0.6%, respectively (Fig 6B). The ratio of the AS types was inconsistent with that reported previously for lotus identified from ESTs [37]. To detect the biological processes in which these AS genes might be involved, the GO term enrichment was investigated (Fig 6C). For biological process, genes involved in the metabolic process and cellular process were highly represented. For molecular function, binding was the most represented GO term, followed by catalytic activity. For cellular component, the major categories were cell and cell part. Some GO terms were not enriched in the AS genes, such as the terms of growth, locomotion and rhythmic process in biological process, auxiliary transport protein in molecular function, and virion part in cellular component.

It has been demonstrated that AS events play an important role in an evolutionary mechanism of posttranscriptional regulation [33–35]. However, the mechanisms of AS in lotus have recently begun being explored and remain poorly understood [37]. In this study, we proposed that the difference in expressed genes between leaves and rhizomes was the result of AS, which might function in a specific manner to regulate gene expression. Further sequencing of additional organisms will facilitate to unravel the effects of genomic features on the mechanisms of splice site recognition.

### Validation of SNPs and AS events

To evaluate the validation rate of the SNPs identified by bioinformatic analysis, a total of 53 SNPs were validated by PCR amplification and direct sequencing. Among the primer pairs designed for validation of these SNPs, primer pairs for 42 SNPs could amplify target sequences. Within these amplified sequences, 31 SNPs were validated in ‘BG’, ‘WR1’, ‘ZO’ and ‘RL’, and other 7 SNPs were identified in ‘Yehong Lian’, ‘Jianxian 17’, ‘Luming Lian’, ‘AL1’, and ‘Golden Bird’. The estimated predicting accuracy reached 74% (Table 4).

**Table 4:** Summary of SNP validation in ‘BG’, ‘WR1’, ‘ZO’ and ‘RL’.

Genes	Location of sequenced fragment	Number of SNPs tested	Number of SNPs validated
Serine-rich-splicing factor RSP31	Scaffold _0: 14017975..14020556	7	7
Histone H4	Scaffold _1:13658354..13663290	5	3
Probable LRR receptor-like serine/threonine-protein kinase (Y4372)	Scaffold _2:944883..944913	11	8
Ferric reduction oxidase 8 (FRO8)	Scaffold _5:1765570..1772132	14	10
Succinate dehydrogenase assembly factor 2 (SDHF2)	Scaffold _7:1171910..1172491	5	3
Total		42	31

**Fig 7:**
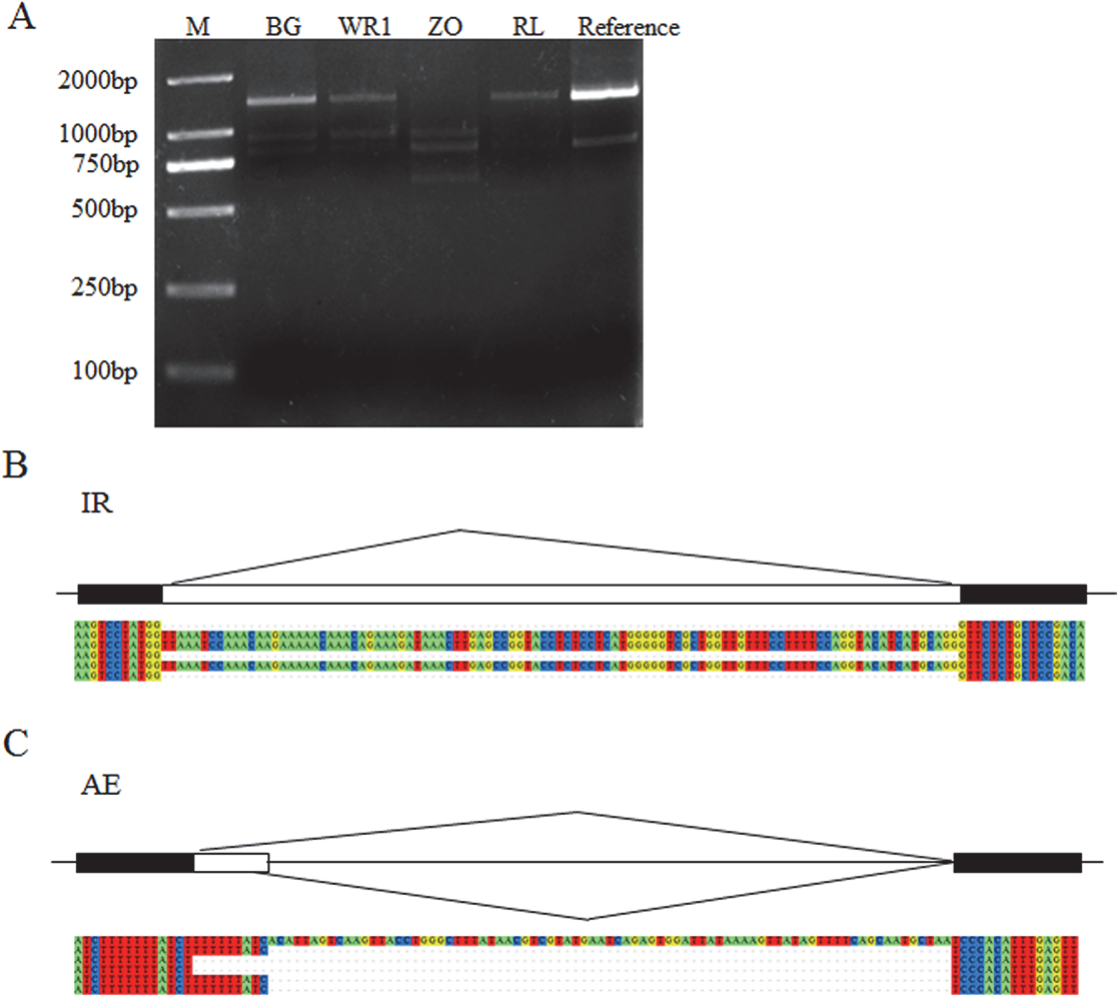
Validation of AS events in the Asian lotus transcriptome. (A) Validation of AS in NNU_25229-RA. (B) Validation of IR in NNU_21858-RA. (C) Validation of AE in NNU_17825-RA.

Seven AS genes were used for validation. Among seven pairs of primers designed, five primer pairs could amplify sequences. One primer pair produced more than one polymorphic band with predicted sizes (Fig 7A), which contained IR and AE. Other four pairs of primers produced one band, and these fragments were sequenced to check the corresponding AS events, including two IR (Fig 7B), one AE (Fig 7C), and one ES. But the ES was not supported by our validation experiments.

## Conclusions

In this study, the transcriptomes of four Asian lotus cultivars were sequenced with Illumina HiSeq2000 platform, and large numbers of SNPs and AS events were uncovered with an assembled reference transcriptome. Our study revealed the complexity of the Asian lotus transcriptome, and gave extensive insights on alternative splicing and gene structure. The results will not only serve to complement the predicted gene database of lotus, but also to provide an invaluable resource for future functional genomic studies on lotus species.

## Supporting Information

S1 Table Primer information for SNP and AS validation. (PDF)
