# Supplementary material for: RNA-Seq Uncovers SNPs and Alternative Splicing Events in Asian Lotus (Nelumbo nucifera)
Source: PLoS One. 2015 Apr 30;10(4):e0125702. doi: 10.1371/journal.pone.0125702 (PMC4416007; doi:10.1371/journal.pone.0125702)
Supplement: S1 Table — (PDF) [file pone.0125702.s001.pdf]

S1 Table. Primer information for SNP and AS validation.

Gene	Validation	Forward primer (5’→3’)	Reverse primer (5’→3’)
RSP31	SNP	GAAGAGGTGGCTACGGGAGG	CGTGGGCATTGAGTTTGAGG
H4	SNP	AAAATCATCACAGGCGAAAT	AGAACCATAGCAGGAAGCAC
Y4372	SNP	CCGAGGTTCTTGTGCTTGAC	TTGGTGGTTGGAGATGTGCT
FRO8	SNP	ATTTCTGCTCGCATCTTCCC	TGTTTCCTTGCTCAGCCTTC
SDHF2	SNP	GGGGTCCGTTTGTTTCGTAG	AACTGGCTGGTCGTCTCATC
NNU_25229-RA	IR and AE	TCCTCTATGAAAGGGATTGC	GTGCTGGGTCGTTTGTAAGT
NNU_21858-RA	IR	CAGGGAGGAGCTAATGTCGT	ATGTCTCGGATTTCAGGTGG
NNU_24367-RA	IR	TGTGCCACTCCGTGCTTATT	ACCTCTGCCGTCCATTCCTC
NNU_17825-RA	AE	AGGTGGTGGATGAGAAGTAT	TGTGGAGTATCGTGGAGTGA
NNU_13286-RA	ES	TGATGGACCAGTAGGAGGAC	ATTAGCCAAGATGCTGAAAA
